# Portable Infrared-Based Glucometer Reinforced with Fuzzy Logic

**DOI:** 10.3390/bios13110991

**Published:** 2023-11-20

**Authors:** Hasan Mhd Nazha, Mhd Ayham Darwich, Ebrahim Ismaiel, Anas Shahen, Tamim Nasser, Maher Assaad, Daniel Juhre

**Affiliations:** 1 Computational Mechanics, Faculty of Mechanical Engineering, Otto Von Guericke University Magdeburg, Universitätsplatz 2, 39106 Magdeburg, Germany; hasan.nazha@ovgu.de (H.M.N.); daniel.juhre@ovgu.de (D.J.); 2 Faculty of Technical Engineering, Tartous University, Tartous P.O. Box 2147, Syria; 3 Faculty of Biomedical Engineering, Al-Andalus University for Medical Sciences, Tartous P.O. Box 101, Syria; ei04@au.edu.sy (E.I.); as12@au.edu.sy (A.S.); tn12@au.edu.sy (T.N.); 4 Department of Electrical and Computer Engineering, College of Engineering and IT, Ajman University, Ajman P.O. Box 346, United Arab Emirates; m.assaad@ajman.ac.ae

**Keywords:** glucose monitoring, mid-infrared probe, fuzzy logic, tears

## Abstract

Diabetes mellitus (DM) is a chronic metabolic condition characterized by high blood glucose levels owing to decreased insulin production or sensitivity. Current diagnostic approaches for gestational diabetes entail intrusive blood tests, which are painful and impractical for regular monitoring. Additionally, typical blood glucose monitoring systems are restricted in their measurement frequency and need finger pricks for blood samples. This research study focuses on the development of a non-invasive, real-time glucose monitoring method based on the detection of glucose in human tears and finger blood using mid-infrared (IR) spectroscopy. The proposed solution combines a fuzzy logic-based calibration mechanism with an IR sensor and Arduino controller. This calibration technique increases the accuracy of non-invasive glucose testing based on MID absorbance in fingertips and human tears. The data demonstrate that our device has high accuracy and reliability, with an error rate of less than 3%, according to the EGA. Out of 360 measurements, 97.5% fell into zone A, 2.2% into zone B, and 0.3% into zone C of the Clarke Error Grid. This suggests that our device can give clinically precise and acceptable estimates of blood glucose levels without inflicting any harm or discomfort on the user.

## 1. Introduction

Diabetes mellitus (DM) is a chronic metabolic disorder that impairs the body’s ability to effectively utilize glucose. Glucose, obtained from food, undergoes metabolic processes and enters the bloodstream, where insulin plays a vital role in facilitating its uptake by cells for energy production [1]. However, individuals with diabetes experience difficulties in insulin secretion or sensitivity, leading to persistently elevated blood glucose levels [2].

Currently, the primary diagnostic methods for gestational diabetes involve blood tests, including the glucose screening test and the glucose tolerance test [3]. Unfortunately, the lack of non-invasive techniques for measuring glucose levels has been a longstanding challenge [4]. The glucose tolerance test, considered the gold standard, can be invasive and uncomfortable for patients as it requires the consumption of a glucose beverage and multiple blood draws [5]. On the other hand, the glucose screening test is less invasive but provides only a snapshot of blood glucose levels at the time of testing [6]. Therefore, there is a critical need to develop non-invasive, real-time glucose monitoring technologies to enhance the accuracy and convenience of diagnosing and managing gestational diabetes [7]. Current blood glucose monitoring devices available on the market offer a limited number of measurements per day, which falls short of the continuous real-time monitoring required by diabetic patients, particularly those who rely on insulin [8]. Moreover, traditional glucometers necessitate finger pricks for blood sampling, causing discomfort and potentially leading to long-term finger damage, which may discourage frequent monitoring [5]. As a result, an ideal continuous glucose monitoring (CGM) device should be non-invasive, portable, accurate, affordable, user-friendly, and minimize the need for frequent calibration. In the quest for non-invasive glucose measurement, mid-infrared (IR) radiation within the wavelength range of 2500 nm to 25 µm has shown promising potential due to its high selectivity for low-concentration compounds found in complex organic media. Techniques utilizing mid-infrared, such as diffuse reflectance spectroscopy and photothermal detectors, leverage the strong water absorption in living tissue to access glucose molecules from the epidermal layer or even deeper depths [6,7,8].

Lilienfeld-Toal et al. [9] conducted a pioneering study that focused on the combined use of photoacoustic (PA) spectroscopy and mid-infrared (MIR) spectroscopy for glucose measurements. In their research, they employed two distinct quantum cascade lasers (QCLs) to generate heat pulses on a human forearm. The first laser operated at a specific wavelength of 1080 cm^−1^, which is known to correspond to the peak glucose absorption. The second laser, on the other hand, was utilized to minimize any interfering background noise caused by significant water absorption at 1066 cm^−1^. To detect the resulting PA signals emitted from the skin, a highly sensitive microphone was placed within an acoustic cell. The obtained correlation value between the PA signals and glucose concentration was determined to be 0.61. In 2011, Pleitez et al. [10] conducted a study that aimed to advance the application of three quantum cascade lasers (QCLs) for palm-based glucose level detection. The study identified three specific infrared wavelengths (1084 cm^−1^, 1054 cm^−1^, and 1100 cm^−1^) to detect glucose peaks, while 1100 cm^−1^ served as the reference background wavelength. To facilitate this, a twin Helmholtz gas cell was utilized as an acoustic cell with a resonance frequency of 2 kHz. Comparatively, the correlation factor (R) of the study was enhanced to 0.7, an improvement over their previous research [10]. Kottmann et al. [11] proposed a silver halide optical fiber that is flexible and non-toxic, allowing for the effective delivery of light to different areas of the body. In an aqueous glucose solution, they achieved a detection limit of 57 mg/dL and a signal-to-noise ratio (SNR) of 1, with a high correlation coefficient (R^2^ = 0.993). Three years later, the same research group utilized a dual-wavelength approach, using 1080 cm^−1^ for the glucose peak and 1180 cm^−1^ for the background. They gathered acoustic signals for glucose detection in vivo from a fasting, healthy volunteer’s forearm and fingertip. The prediction limit was enhanced to ±30 at a 90% confidence level for glucose concentrations ranging from 90 to 170 mg/dL. Aloraynan et al. [12] created a photoacoustic device for noninvasive glucose monitoring that uses a single-wavelength quantum cascade laser with a glucose fingerprint of 1080 cm1. The technology was tested on artificial skin phantoms with normal and hyperglycemia blood glucose values. The system’s detection sensitivity has been increased to 25 mg/dL, utilizing a single wavelength for the whole range of blood glucose. Machine learning has been used to identify glucose levels in skin samples using photoacoustic spectroscopy. Using classification approaches, ensemble machine learning models have been constructed to assess glucose concentration. The model obtained a prediction accuracy of 90.4%, with 100% of the projected data falling into Clarke’s error grid analysis zones A and B.

The measurement of glucose based on human tears has gained significant attention in recent studies as a potential non-invasive method for glucose monitoring in individuals with diabetes. A study conducted by Aihara et al. [13] explored the correlation between tear glucose levels and blood glucose levels using a tear glucose analyzer. The researchers found a strong positive correlation between tear glucose and blood glucose levels, suggesting that tears can serve as a reliable indicator of blood glucose concentration. Another recent study by Kim et al. [14] investigated noninvasive glucose monitoring using nanoparticle-embedded contact lenses (NECL). By analyzing changes in the reflection spectrum of the contact lenses, a correlation curve was established to determine glucose concentration. The study demonstrated the potential of NECL as a biocompatible biosensor for regular monitoring of tear glucose levels, offering a simple and noninvasive approach to glucose monitoring. These studies highlight the potential of tear-based glucose measurement as a non-invasive and convenient approach for monitoring blood glucose levels in diabetic individuals, paving the way for future advancements in tear-based glucose monitoring technologies.

However, most non-invasive devices still require frequent calibration, which can be impractical and ineffective. Recent research endeavors have focused on reducing or eliminating the need for frequent calibration [15,16]. In this context, the introduced approach presents a novel method by utilizing a fuzzy logic-based calibration system that incorporates an IR sensor and Arduino controller. This system accurately correlates the output voltage of the sensor with reliable glucose concentrations, employing the Clarke Error Grid to estimate error tolerance based on the output voltage and estimated glucose concentration from the fingertip and tears. Additionally, this approach significantly improves the accuracy of non-invasive glucose measurement based on the composition of human tears and the blood flow of the finger.

The paper is organized as follows: Section 2 presents a brief overview of the near-infrared (NIR) and its behavior in liquids, especially water. Section 3 illustrates the methods and materials that have been utilized and developed to achieve the measurement system. Section 4 addresses the results and provides a comprehensive discussion about the findings and related work.

## 2. Theoretical Background

Light rays are subjected to many phenomena as they pass through the tissues of the human body, including scattering, absorption, reflection, and refraction. These occurrences are indications of irreconcilable refraction and reflection between the interior and outside of the cell, as well as through the fluid. The detection should be practically constant in theory, but it may fluctuate when the concentration of glucose molecules varies, and the Bouguer–Lambert law indicates that the quantity of light absorbed by a material depends on the concentration and length of the path the light takes. When compared to less dense tissues, increased absorption in tissues containing numerous sugar molecules lowers optical density through such tissues. A wavelength of 940 nm has been proven to be therapeutically acceptable due to its low absorption and the fact that the intensity of light traveling through blood vessels is not diminished due to sugar molecule absorption [6]. After absorption, the resultant optical density relationship is given by Equation (1):

(1) $$I=I_{0}e^{-\mu_{eff}L}$$

where:

*I* is the corresponding optical density;

*I*_0_ is the transmitted optical density;

*L* is the length of path crossed by light;

$\mu_{eff}$ is the coefficient of loss within tissue, defined by Equation (2):

(2) $$\mu_{eff}=\sqrt{2\mu_{a}\left(\mu_{a}+\mu_{s}’\right)}$$

where $\mu_{a}$ and $\mu_{s}$(mm^−1^) are the tissue optical properties representing the absorption and the scattering coefficients.

After being processed and calibrated, a change in light density after absorption shows as a change in voltage, which leads to a change in the output presented as blood glucose percentage.

The gaseous state of the water molecule contains three distinct types of transitions that might result in electromagnetic radiation absorption. The absorption of atmospheric water vapor in the far-infrared region of the spectrum is caused by rotating motions in which the molecule obtains extra rotational energy. This absorption occurs at wavelengths ranging from about 200 cm^−1^ (50 m) to longer wavelengths, extending into the microwave domain. The process through which a molecule gains an increase in vibrational energy is referred to as a vibrational transition. Absorption in the mid-infrared region, notably at 1650 cm^−1^ (known as the μ band, with a wavelength of 6 μm) and 3500 cm^−1^ (referred to as the X band, with a wavelength of 2.9 μm), may provide evidence for these transitions. When a molecule is stimulated to an excited electronic state, electronic transitions occur. In this category, the transition with the least energy is observed in the vacuum ultraviolet region.

## 3. Materials and Methods

### 3.1. Hardware Design

#### 3.1.1. IR Transmitter and Receiver

We employed infrared sensors to assess the sugar levels in blood vessels. These sensors have the benefit of producing an analog voltage output proportional to the amount of light received. An infrared sensor is made up of a sensor and a photoreceptor that transforms the incoming train of IR pulses into an analog output signal that we then utilize as input for the Arduino Uno platform (Arduino UNO R3, ATmega 328 AVR controller, Microchip Technology, Arduino AG, Milan, Italy). The transmitter and receiver were derived from the very sensitive TP808 photocoupler element, which consists of infrared diodes and NPN phototransistors with a high sensitivity of 980 nm wavelength and 30 mW. The NIR measurements are driven by a PWM (Pulse Width Modulation) signal of a fixed frequency of 1 kHz. This was achieved to ensure stability and precision. We implemented a transimpedance operational amplifier (op-amp) structure with a 300 kΩ resistor (R_f_) to convert the output current to voltage. This configuration is a well-established and widely used approach for current-to-voltage conversion in various applications [17].

#### 3.1.2. Amplifiers and Filters

The Lm358 amplifier was used for this purpose. A non-inverting amplifier formula has been used that calculates the gain from the following relationship (3):

(3) $$G=1+\frac{R_{2}}{R_{1}}$$

where *R*_1_ and *R*_2_ are the resistors used to determine the gain of the amplifier.

A passive low-pass filter *RC* (*C* = 10 µF, *R* = 330 Ω) has been connected after the amplifier to remove unwanted signals such as power supply noise.

#### 3.1.3. Final Monitoring Circuit

**Figure 1:**
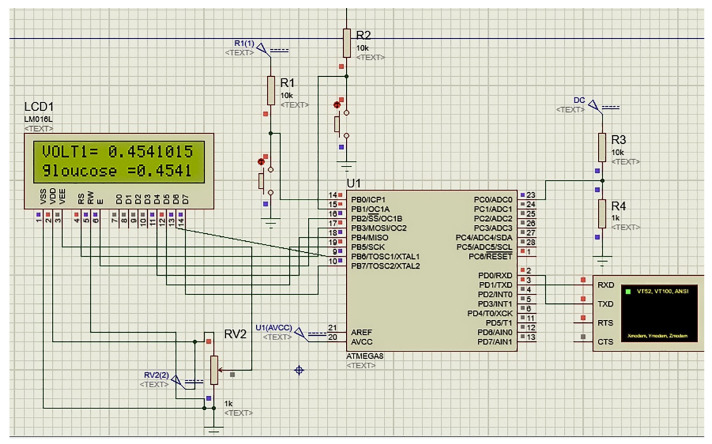
The simulated portable glucose monitor in this study using Atmega8 controller.

The implemented infrared monitoring system (Figure 1) was created by combining the IR circuit (see Section 3.1.1) with the Arduino Uno platform to transform the analog input from the IR sensor into digital information.

#### 3.1.4. Measurement Procedures

The measurement procedures in our study comprised two pivotal aspects: the technological setup, which entailed attaching an infrared emitter and detector to the fingertip, and the meticulous collection of tears from the participants. In this section, we provide a detailed account of these procedures, along with relevant citations to highlight the methodology’s robustness.

For the technological setup, we followed a method previously described by Ogunsanya et al. [18], where an infrared emitter and detector were securely affixed to the fingertip. This setup allowed us to non-invasively measure blood glucose levels by analyzing the changes in light absorption through the fingertip tissue. Our goal was to ensure precision and consistency, which is why we meticulously described the step-by-step procedure for the sensor attachment in the methodology section.

Simultaneously, our methodology incorporated the collection of tears, a vital component of our study. Tears are known to contain biomarkers related to glucose levels, as discussed in Aihara et al. [13]. To gather tears, we followed a standardized protocol derived from the Tear Collection Guidelines published cited in Bachhuber et al. [19]. This protocol ensured the reliability and accuracy of the data we obtained, making our tear collection process robust and consistent.

To assess the performance of our circuit, we conducted a preliminary investigation involving 30 participants, comprising 15 individuals with diabetes and 15 healthy controls. The study encompassed the assessment of their blood glucose levels using our approach and a reference device following the conventional finger-prick method. Measurements were taken both before and after meals at 15 min intervals, resulting in a total of 12 measurements per participant. Each trial began with measuring glucose using a Metene TD-4116 Blood Glucose Monitor portable device (reference device), as commonly practiced in diabetic research [11]. Subsequently, our custom-designed device was tested for its glucose monitoring capabilities.

### 3.2. Fuzzy Logic with CEG and Tears

**Figure 2:**
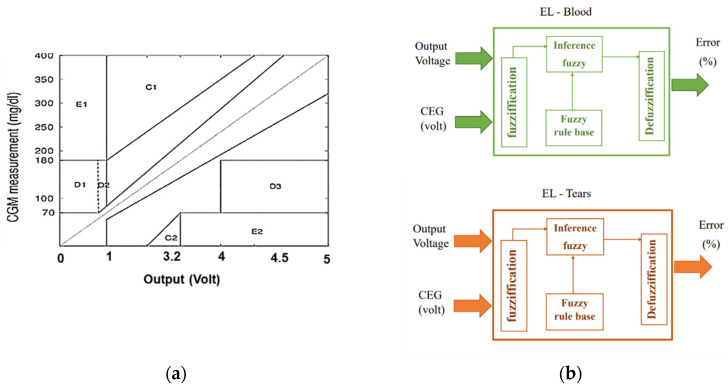
Proposed methods for mapping Arduino voltage to glucose levels and determining error tolerance using FL: (**a**) Clarke grid with voltage output on the horizontal axis; (**b**) suggested fuzzy logic with voltage and CGM inputs and error output.

The Clarke Error Grid (CEG), which has been authorized for clinical use [20], was utilized to identify disparities between test glucose measurement methodologies and baseline intravenous blood glucose readings. In this study, the horizontal axis of CEG (Figure 2a) represents the output voltage of the Arduino based on the preliminary calibration procedure, while the Y-axis represents the values obtained from the reference device. The labeled region is a perfect match between the two. Region-A (acceptable) glucose readings differ from the 20% reference value or are within the blood glucose range (70 mg/dL). Values within this range are clinically accurate, resulting in the proper clinical diagnosis. Region-B (benign flaws) is located above and underneath Region-A. This range shows values that differ from the baseline by 20%. Zones A and B are clinically acceptable; however, values within areas are also presented. Regions C and E have the potential to be problematic and cause clinically substantial mistakes.

The major goal of this approach is to determine the predicted error tolerance of glucose concentration, which represents the confidence interval of the calculated glucose value. FL (Figure 2b) was used to assess the output voltage from Arduino and the average accuracy of the measured voltage based on CEG’s regions (on a scale of 0 to 1) to estimate the inaccuracy of the observed glucose. A similar method was used with tears to measure glucose tolerance.

**Figure 3:**
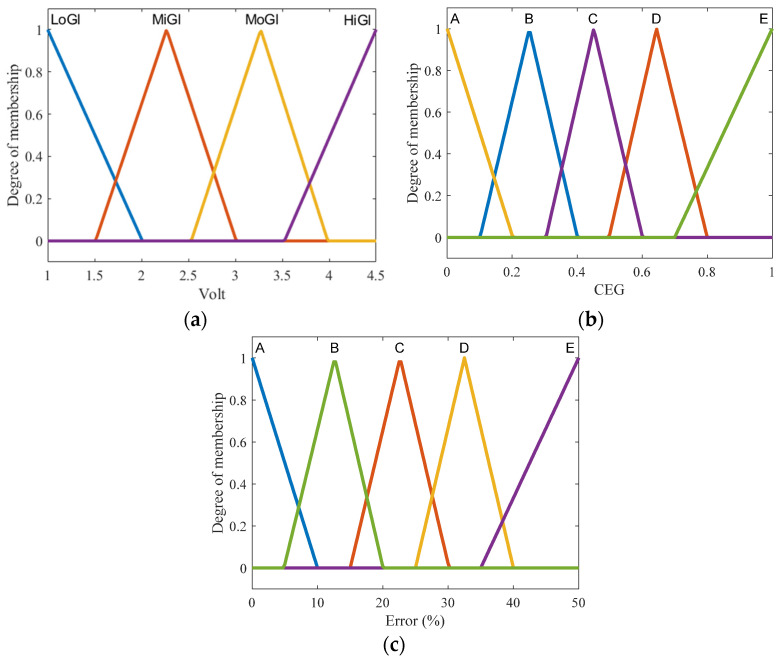
Membership functions of input and output in the proposed FL system: (**a**) analog-voltage input MFs; (**b**) CEG input MFs; (**c**) FL output MFs.

The fuzzy system was built based on the measurements of the CGM versus the actual output voltage of the designed device. An FL model including the MFs and fuzzy rules was designed based on the experimental results from this study. The intrinsic concept of using FL is to predict the error of the estimated glucose based the voltage without the need of CGM or the reference device. The FL system involves multiple stages to get the expected error for each fingertip and tear measurement, as follows:

1. Fuzzification entails creating membership functions (MF) for the input variables in order to determine the degree of truth in each rule. Based on the regions of CEG, the input has two variables: Arduino output and voltage accuracy. The MFs of voltage output are shown in Figure 3a, where the MFs of output voltage are represented by two triangle functions labeled low glucose (LoGl) and high glucose (HiGl), as well as two triangle functions labeled mild glucose (MiGl) and moderate glucose (MoGl). The second input consists of MFs with (A-E) labels that indicate CEG zones (Figure 3b) as the normalized position of the voltage point in each region (where 0 is close to region A, and 1 is closer to region E). The MFs of the output are similar to the second input, but the actual values as % of error vary between 0 and 100 (Figure 3c).

2. Inference includes fuzzy if-then rules. In this work, fuzzy rules were developed based on experimental observations of glucose and its locations on Clarke grid regions. Some examples of fuzzy rules include the following:

“If Voltage is LoGl and CEG is A then Error is A”;

“If Voltage is LoGl and CEG is B then Error is B”.

3. Defuzzification describes the process of converting the fuzzy value back into the real one. Here, the “centroid” method, which depends on the center of gravity, was used to obtain the final output, which is the error percentage of glucose concentration.

## 4. Results

### 4.1. Glucose Acquisition system

Non-invasive blood glucose monitoring using IR light is regarded as a convenient and dependable technology for measuring blood sugar levels during regular activities. The principle of IR-based glucose monitoring is based on the variable absorption levels of IR light by blood with high or low levels of glucose solution.

We performed a pilot study on 30 patients, 15 of whom had diabetes and 15 of whom were healthy controls, to evaluate our gadget. We used our gadget and a reference device that employs the usual finger-prick approach to assess their blood glucose levels.

Our proposed system, which used a 940 nm IR light source, seemed to be capable of producing discernible signals. As indicated in Table 1, the recorded signals seem to differ across people, particularly between normal (1.22–2.25 volts) and diabetic (2.9–3.5 volts) patients. These findings are consistent with the findings of Yunos et al., who preferred the use of the IR principle to boost measurement sensitivity and highlighted the accuracy of this approach in discriminating between distinct groups of potential patients [21].

**Figure 4:**
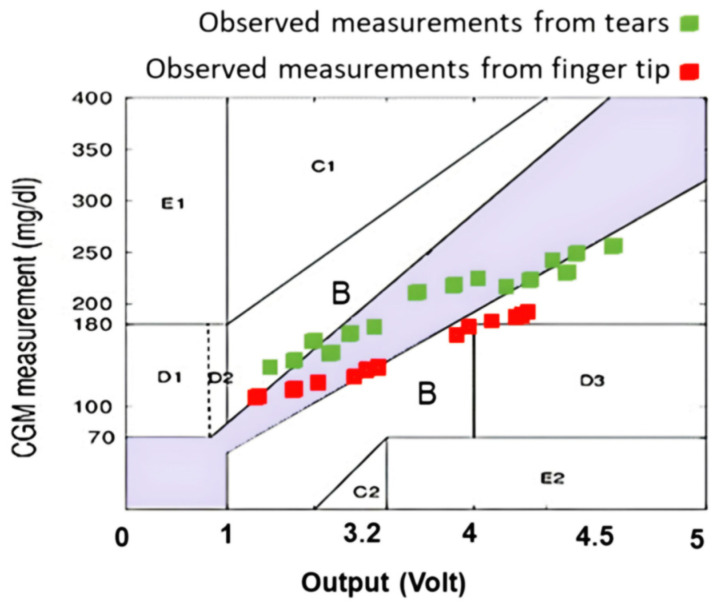
Measurements were graphed on the Clarke grid using the recordings from the Arduino and reference device.

The results in Table 1 represent the final measurements, which are also presented in Figure 4. The values demonstrate a less-than-satisfactory convergence in the “A” and “B” sections, indicating an inaccuracy in the predicted glucose level, necessitating the use of FL to calculate the anticipated tolerance. We considered the measurement of the reference device as the raw CGM value. In this study, the CGM is considered to be the actual and precise values that should be used for the calibration and validation stages.

### 4.2. Glucose Measurement Using FL

Table 2 shows that FL might be proposed for error mapping and validation. By establishing the predicted error, the estimated tolerance of glucose utilizing FL output provides a more trustworthy measurement of the monitor system. Glucose levels in healthy samples range between 104 and 115 mg/dcL, with an inaccuracy of less than 10%. Glucose errors in diabetic samples range between 5% and 10%. Since the FL model aims to predict the error of recording values, only the (±standard deviation) value represents FL output, while the average values in the “Glucose using FL” column of Table 2 are the same values as those in the “Glucose using Device” column.

The findings showed that using NIR with fingers and tears in parallel may test glucose in a non-invasive and painless way while maintaining adequate accuracy and reliability. The outcomes of the proposed FL were systematically assessed by computing the standard deviation of the 12 measurements per participant recorded from both reference and proposed devices. These values were then compared to the predicted error from the FL, as indicated in Table 3. The error values between the devices and the proposed FL model demonstrate similar error margins relatively between the predicted and actual results among the repeated measurements of the same participants. 

## 5. Discussion

Our study was initially grounded in the concept of establishing a correlation between blood glucose values and the corresponding values recorded from tears. While we acknowledge the time delay between blood glucose and tear glucose levels, our approach aligns with emerging research in the field. Recent studies, such as those by [22,23], have explored this relationship and found promising results, emphasizing the potential of tears as a non-invasive medium for glucose monitoring.

Moreover, to address the potential shift in measurements, we conducted thorough material testing by applying the suggested continuous glucose monitoring method to both normal and diabetic individuals, as suggested by [24]. This comprehensive testing allowed us to assess the impact of any experimental, hardware, or logical flaws, which have extensively been discussed in our revised manuscript.

Peters et al. [25] has demonstrated that the optical properties of blood and tissues are sensitive to changes in glucose concentration, making it possible to correlate changes in these properties with blood glucose levels. The choice of the 940 nm wavelength is based on its suitability for glucose monitoring. It falls within the near-infrared region, where the absorption of glucose is sensitive to changes in its concentration. This wavelength has been widely used in non-invasive glucose monitoring studies [26]. While it may not seem intuitive, the choice of this wavelength is supported by its ability to provide accurate estimates of glucose levels.

The parallel measurement of blood glucose levels using both finger and tear samples in our study is a pivotal aspect of our methodology. However, the process of integrating these measurements to derive the final estimation of blood glucose lacks a detailed exposition in the current presentation. To address this gap, it is essential to provide a thorough description of the data processing techniques applied, highlighting how the data from both measurements are harmonized. By doing so, we can better elucidate the specific contributions of each measurement to the overall accuracy of the blood glucose estimation.

Numerous studies have explored the integration of multiple data sources to improve the accuracy of blood glucose estimations. For instance, Xiong et al. [27] demonstrated the efficacy of a dual-source data integration model using both continuous glucose monitoring and self-monitoring of blood glucose. This approach showcased significant improvements in accuracy when compared to single-source measurements. Therefore, considering the insights from studies like this, it becomes crucial to expound on the data fusion process in our research and its potential to enhance accuracy.

Furthermore, it is vital to discuss the potential implications of relying solely on one of the two measurements. Prior research, such as the work by Tolks et al. [28], has explored the impact of using a single measurement source on the accuracy of blood glucose estimation. These findings underscore the importance of elucidating the accuracy reduction that might occur when only one of the two measurements is employed. Addressing these aspects in our discussion will provide a more comprehensive understanding of the strengths and limitations of our approach. This outcome is consistent with Mehmood et al.’s work [28], which revealed encouraging results with the application of fuzzy logic in artificial pancreas control schemes.

In this paper, we attempt to present the concept of FL-based Clarke error grids. An infrared emitter and detector mounted to the fingertip and coupled to an Arduino microcontroller comprise the finger probe. Many studies have advocated the use of fuzzy logic for measuring glucose levels [29,30,31]. The FL algorithm seemed to assess the accuracy and reliability of the glucose test using the Clarke error grid at an acceptable rate. It offered a graphical approach for comparing various glucose measurement techniques. By separating the measurement area into five zones: A, B, C, D, and E, we were able to categorize the measurements from the most accurate to the most erroneous, resulting in harmful results. Fuzzy logic was used to describe measurement mistakes in a flexible and realistic manner. The FL algorithm was able to obtain more accurate glucose readings by assigning membership to each zone of the Clarke error grid as a new utilization of FL compared to [30]. Numerous strategies, such as photoacoustics [9,10,11,12], and technologies that use radiation in the near- or mid-infrared [6,7,8], were proposed. When compared to techniques using radiation in the near-infrared regions, methods utilizing radiation where glucose exhibits significant absorption are beneficial for increasing the measurement’s sensitivity. The poor measurement accuracy of these noninvasive blood glucose measuring methods presents a fundamental challenge for their practical application, so measurement performance is extremely important, particularly when used in real-world settings.

By identifying which emission depends on the change in blood glucose and which is connected to interstitial glucose, and then evaluating a correction model, it may be possible to obtain more precise readings because this method can detect emissions from depths of about 1.2 mm. We will broaden the scope of our studies to include more participants and gather data from measurements of subjects other than the human body for additional validation in order to achieve remote glucose measurement by mid-infrared passive spectroscopic imaging. It is especially important to carefully and thoroughly investigate how electrolytes, proteins, water, physiological cues, and potential environmental noise affect the depth of readings.

## 6. Conclusions

Non-invasive blood glucose monitoring is a desired objective for many diabetic individuals who need to detect their glucose levels regularly and correctly. However, most of the present techniques are either intrusive or require costly and specialized equipment. Fuzzy logic is a mathematical approach that can manage uncertainty and imprecision in data and has been used to address numerous health challenges, such as artificial pancreas control and glucose testing utilizing heart rate variability. FL may also be utilized to construct simple and resilient measuring devices that can adjust to changing physiological circumstances and environmental influences.

In this study, we offered a contribution relating to the use of FL measurements to estimate the blood glucose level from the fingertip and tears in parallel. To test our technology, we performed a preliminary study on 30 patients, comprising 15 people with diabetes and 15 healthy controls. We tested their blood glucose levels using our technology and a reference device that employs the usual finger-prick approach. Measurements were acquired and recorded before and after a meal, at intervals of 15 min, for a total of 12 measurements per person. We next compared the FL results to the reference readings using the Clarke error grid analysis, which is a commonly accepted approach to assessing the accuracy of glucose meters.

The findings reveal that our gadget has high accuracy and dependability, with an error rate of less than 3%, according to the EGA. Out of 360 measurements, 97.5% fell into zone A, 2.2% into zone B, and 0.3% into zone C. No readings fell within zones D or E. This implies that our gadget can deliver clinically accurate and acceptable estimations of blood glucose levels without causing any injury or pain to the user.

We conclude that our technology is a potential alternative to invasive technologies for blood glucose monitoring, particularly for diabetic patients who require regular assessments. Our device employs FL measurements to estimate the blood glucose level from skin impedance, which is a straightforward and non-invasive approach that may be applied to a portable device. Our gadget has high precision and dependability, with an error rate of less than 3%, according to the Clarke error grid analysis. This way of employing FL with a Clarke error grid appears to produce a more confident and exact output for this sort of portable instrument.

## Figures and Tables

**Table 1:** Glucose levels and associated recorded signal values of 15 participants.

Glucose ^1^ (mg/dcL)	Output (Volt) of Finger Tip	Output (Volt) of Tears	Age	Glucose Using Device ^2^ (mg/dcL	Gender	Diabetes (Neg/Pos)	Fasting
110	1.55	1.68	16	116	M	Neg	No
115	1.75	2.09	24	121	M	Neg	No
104	1.25	1.63	24	96	M	Neg	No
167	3.2	3.52	65	158	F	Pos	Yes
126	2.15	2.35	23	138	M	Neg	No
128	2.25	2.54	23	125	M	Neg	No
120	2.05	2.34	23	113	F	Neg	No
103	1.22	1.3	25	99	M	Neg	Yes
109	1.53	2.05	23	102	F	Neg	No
115	1.74	1.84	24	109	M	Neg	No
162	3.01	3.06	45	175	F	Pos	Yes
170	3.4	4.31	50	161	M	Pos	Yes
155	2.9	3.5	48	168	M	Pos	Yes
172	3.45	4.36	45	166	F	Pos	Yes
175	3.5	4.46	58	178	M	Pos	Yes

^1^ Using reference device. ^2^ Calculated glucose using voltage output.

**Table 2:** A comparison of 15 participants’ measured glucose levels with the referential device and FL-based error.

Glucose ^1^ (mg/dcL)	Output (Volt) Using Finger Tip	Output (Volt) Using Tears	Glucose Using Device ^2^ (mg/dcL)	Glucose Using FL ^3^
110	1.55	1.68	116	116 ± 6
115	1.75	2.09	121	121 ± 6
104	1.25	1.63	96	96 ± 8
167	3.2	3.52	158	158 ± 9
126	2.15	2.35	138	138 ± 12
128	2.25	2.54	125	125 ± 3
120	2.05	2.34	113	113 ± 7
103	1.22	1.3	99	99 ± 4
109	1.53	2.05	102	102 ± 7
115	1.74	1.84	109	109 ± 6
162	3.01	3.06	175	175 ± 13
170	3.4	4.31	161	161 ± 9
155	2.9	3.5	168	168 ± 13
172	3.45	4.36	166	166 ± 6
175	3.5	4.46	178	178 ± 3

^1^ Using reference device. ^2^ Calculated glucose using voltage output. ^3^ Calculated glucose with possible error using FL.

**Table 3:** The average and standard deviation (SD) measurements per participant were obtained from the reference, proposed devices, and the FL model. The latter was used only to predict the SD of the proposed device’s measurements.

Glucose ^1^ (mg/dcL)	Glucose Using Device ^2^ (mg/dcL)	FL ^3^
110 ± 3	116 ± 8	116 ± 6
115 ± 2	121 ± 7	121 ± 6
104 ± 4	96 ± 6	96 ± 8
167 ± 3	158 ± 8	158 ± 9
126 ± 6	138 ± 11	138 ± 12
128 ± 3	125 ± 4	125 ± 3
120 ± 4	113 ± 7	113 ± 7
103 ± 3	99 ± 5	99 ± 4
109 ± 5	102 ± 8	102 ± 7
115 ± 3	109 ± 5	109 ± 6
162 ± 6	175 ± 14	175 ± 13
170 ± 5	161 ± 11	161 ± 9
155 ± 5	168 ± 11	168 ± 13
172 ± 3	166 ± 3	166 ± 6
175 ± 3	178 ± 4	178 ± 3

^1^ Using reference device. ^2^ Calculated glucose using voltage output. ^3^ Calculated glucose with possible error using FL.

## Data Availability

The datasets generated and/or analyzed during the current study are available from the corresponding author upon reasonable request.
